# Association between sociodemographic determinants and health behaviors, and clustering of health risk behaviors among 28,047 adults: a cross-sectional study among adults from the general Norwegian population

**DOI:** 10.1186/s12889-023-15435-y

**Published:** 2023-03-22

**Authors:** Silje Bjørnerud Korslund, Bjørge Herman Hansen, Tormod Bjørkkjær

**Affiliations:** 1 Department of Nutrition and Public Health, Faculty of Health and Sport Sciences, University of Agder, Kristiansand, Norway; 2 Department of Sport Science and Physical Education, University of Agder, Kristiansand, Norway; 3 Centre for Lifecourse Nutrition, Norway, University of Agder, Kristiansand, Norway

**Keywords:** Adults, Clustering, Socioeconomic factors, Health risk behaviors, Health behaviors, Physical activity, Diet, Tobacco, Alcohol

## Abstract

**Background:**

Understanding the associations between health behaviors and which subgroups are at risk of developing health risk behaviors is vital knowledge to develop effective public health interventions to reduce the high prevalence of non-communicable diseases (NCDs). The objective of the study was to assess the association between physical activity, diet, tobacco use, and alcohol consumption and sociodemographic determinants (sex and education), and to examine clustering patterns of these health behaviors.

**Method:**

Data was collected from an online self-reported questionnaire from the Norwegian public health survey conducted in 2019. The study sample consisted of 28,047 adults (≥ 18 years old) from Agder county in Southern Norway. Chi-square tests and logistic regression analysis were used to determine the association between sex and education according to physical activity, diet, tobacco use and alcohol consumption. Linear regression was used to examine the association between educational level and number of health risk behaviors, and cluster analysis were performed to determine cluster patterns.

**Results:**

Females were more likely than men to meet the national public health recommendations for diet (p < 0.001), tobacco use (p < 0.01), and alcohol consumption (p < 0.001). High education was associated with meeting the recommendations for each of the four health behaviors and with a lower risk of having three or four health risk behaviors simultaneously. Furthermore, clustering of health risk behaviors was observed in five of the sixteen health behavior patterns.

**Conclusion:**

Our findings show a higher risk of having multiple health risk behaviors for males and individuals with low education, and these subgroup findings could inform public health policy and be target goals in future public health interventions. Clustering patterns were observed in over 30% of the health behavior patterns. More research is needed on the causal relationship between health behaviors and socioeconomic factors, and the association between clustering and health outcomes to design effective interventions in the future.

## Background

Non-communicable diseases (NCDs) are the leading cause of death both nationally and globally [1, 2]. Strikingly, more than 40 million people globally die of NCDs yearly, in a time of global pandemic with almost 6.5 million deaths (as of 2 September 2022) due to Covid-19, showing the global pandemic-like burden of NCDs [1–3]. Physical inactivity, unhealthy diet, tobacco use, and high alcohol consumption are well-documented modifiable determinants of health, and central contributors to the development of NCDs such as e.g., cancer, cardiovascular disease, chronic respiratory disease, and type 2 diabetes [2, 4, 5]. The high global incidences of these behaviors are of public health concern, and interventions targeting such behaviors are therefore crucial to promote a healthy lifestyle. Studies have further shown that health behaviors appears to be socially graded with higher prevalence in individuals with lower level of education [6–8].

Although health risk behaviors most often do not occur independently of each other, the majority of previous studies have examined the prevalence of single or pairs of health risk behaviors and their association with health outcomes [9–13], and only a modest body of evidence exists on the prevalence of multiple health risk behaviors, how they cluster across strata of social position and how this clustering is associated with health [14–17]. To our knowledge, there is a scarcity of recent data on the clustering of multiple health risk behaviors in the Norwegian population [18, 19]. Such knowledge is important as multiple health risk behaviors have been shown to have synergistic effects on disease risk, meaning combinations of health risk behaviors are more harmful to an individual’s health than can be expected from the added individual effect alone [18, 20–22]. Further, a previous study found that individuals with low physical activity level, unhealthy diet, tobacco use, and high alcohol consumption reduced their longevity with 14 years, compared to individuals with none of these health risk behaviors [23].

To inform the development of public health initiatives, knowledge on the prevalences of health risk behaviors and how they cluster is vital. Our study aims to investigate the association between sociodemographic determinants and health behaviors according to national public health recommendations, and further explore clustering patterns in a large Norwegian sample of adults. The use of national public health recommendations when we categorize health behaviors is important because it links the analysis of multiple health behaviors to public health goals.

## Methods

### Design, study population and recruitment

This cross-sectional study utilizes data from a Norwegian public health survey from Vest-Agder and Aust-Agder Counties in Southern Norway, conducted between September 23rd and October 18^th,^ 2019, by the Norwegian Institute of Public Health (NIPH) [24]. The survey is designed to collect information about health and living conditions in the population at specific time points in different counties to investigate factors influencing public health. A random sample of 75,191 potential participants (≥ 18 years old with a valid birth number) from all 30 municipalities in Southern Norway was drawn from the National Population Register (31.6% of the adult population in this region). Participants could not be a commuter or a client at an institution. The list of potential participants was validated against the Contact and Reservation Register, and individuals with unverified contact information and individuals who actively reserved against participation in surveys, was removed from the sample (a total of 10,862 individuals). After further removal of deceased individuals and individuals who had not registered an address in Agder Counties, a total of 61,611 potential participants were invited to participate in the survey through SMS and e-mail. Information about the study was promoted through social media, local newspapers, and television. To further increase the participation rate, six random participants each received a gift card worth NOK 4000 (approx. EUR 400). A total of 61,611 participants were invited, and 28,047 responded to the survey and completed the web-based questionnaire, corresponding to a response rate of 45.5% (14,925 females and 13,122 males).

NIPH was responsible for collecting and anonymizing data. All participants provided written, informed consent before participating in the survey. Participation was voluntary, and they could choose to withdrawat any time. The collected data was stored and processed by applicable privacy rules. The study was conducted in line with the Declaration of Helsinki and reviewed by the Faculty Ethical Committee (FEK) at the University of Agder.

### Sociodemographic and behavioral variables

Questions, response alternatives, and variable definitions are presented in Table 1. The socio-demographic variables sex (male/female; recorded from national birth registry), age (from national birth registry), and self-reported education level were included in the study. Age was divided into five categories (18–24, 25–44, 45–66, 67–79, and 80+). Education level was divided into three classes (low, middle, and high education) representing elementary school (10 years), high school (12–13 years), and college degrees less or higher than 4 years, respectively.

**Table 1:** Questions, response alternatives, and variable definitions

Questions	Response Alternatives	Variable Definitions
***Educational level***		
What is your highest completed education?	Primary school/folk high school up to 10 years, vocational training/high school minimum 3 years, college/university less than 4 years, college/university 4 years or more	Low education (reference) Middle education (13 years of education) High education (14 + years of education)
***Age***		
Retrieved from the Central Population Register	≥ 18 years old	Continuous variable (reference; low)
***Physical activity***		
On an average, how often do you exercise in your leisure time?	Never, less than 1 time per week, 1 time per week, 2–3 times per week, 4–5 times per week, each day	High physical activity (≥ 150 min moderate intensity or ≥ 75 min high intensity / week) Low physical activity (reference)
On an average, how hard do you exercise?	Calm without getting out of breath or sweating, getting out of breath and sweating, takes me completely out	
On an average, how long do you exercise each time?	Less than 15 min, 15–29 min, 30 min-1 h, more than 1 h	
***Diet***		
How often do you eat fruit and berries (do not include juices)?	Rarely/never, 1–3 times each month, 1 time per week, 2–3 times per week, 4–6 times per week, daily	High consumption (both fruit and vegetables daily and fish ≥ 2 times / week) Low consumption (reference)
How often do you eat vegetables, included salads (do not include potatoes)?		
How often do you eat fish (as topping on bread, for lunch or dinner)?		
***Tobacco***		
How often do you smoke?	Daily, sometimes; not now, but daily in the past; not now but sometimes in the past; have never smoked	Non-smoker (never, not now but in the past) Smoker (reference)
***Alcohol***		
Have you ever drunk any kind of alcoholic beverage?	Yes, no.	High alcohol consumption (a total score of ≥ 5) Low alcohol consumption (reference)
Within the last 12 months, how often have you had a drink containing alcohol?	Never, ≤ once / month, 2–4 times / month, 2–3 times / week, ≥ 4 times / week	
How many drinks containing alcohol do you have on a typical day of drinking?	1–2, 3–4, 4–5, 5–6, 7–9, ≥ 10	
How often do you have six or more drinks on one occasion?	Never, ≤ once / month, monthly, weekly, daily or almost daily	

Four self-reported health behaviors (physical activity, diet, tobacco use and alcohol consumption) were included in this study.

#### ***Physical activity:***

participants were asked three questions about intensity, duration, and frequency to estimate the participants’ average physical activity level. Moderate intensity was defined as “calm without getting out of breath or sweating” or “getting out of breath and sweating”. High intensity was defined as “takes me completely out”. Duration: the answer “less than 15 min” counted as 8 min, “15–29 min” as 22 min, “30 min-1 hour” as 45 min, and “more than 1 hour” as 75 min. Frequency: the answer “never” counted as 0, “less than 1 time per week” as 0.5, “1 time per week” as 1, “2–3 times per week” as 2.5, and “4–5 times per week” as 4.5 times per week. Further, a combination of these three questions were used to see which participants met the national public health recommendations; at least 150 min of moderate activity or 75 min of vigorous activity per week [25].

#### ***Diet:***

participants were asked three separate questions about their frequency of fish, fruit, and vegetable consumption. These questions regarding diet were further combined into one strict variable, and the participants had to consume fish at least ≥ 2 times per week [26] and have a daily consumption of both fruit and vegetables at the same time to meet the recommendations for diet.

#### ***Tobacco use:***

participants who were former smokers or non-smokers were dichotomized as meeting the recommendations [14].

#### ***Alcohol consumption:***

participants were asked if they had ever drunk alcohol, answering yes or no. Further, three questions from the Alcohol Use Disorders Identification Test (AUDIT-C) [27] were used for those who answered “yes” according to the first question. These questions regarding alcohol consumption included frequency, amounts, and high episodic alcohol consumption, and were used to calculate a score between 0 and 12. Higher scores indicate a higher risk of harmful alcohol use. In the present study, participants scoring ≤ 4 points were categorized as meeting the recommendations [28, 29].

Last, having a health risk behavior is described as not meeting the recommendations for one of the four health behaviors used in this study.

### Statistical analysis

**Fig. 1:**
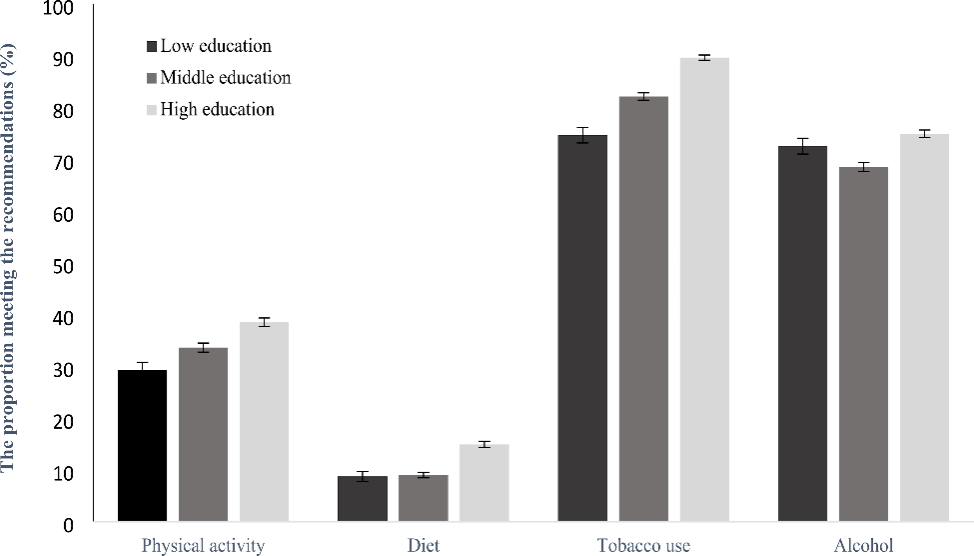
Occurrence of health behaviors (physical activity, healthy diet, non-smoking, and low alcohol consumption) by the level of education (%) with 95% confidence interval)

To determine the differences in physical activity level, diet, tobacco use and alcohol consumption, and the number of health risk behavior between men and women, Pearson chi-square tests were conducted (Table 2). Logistic regression was used to explore the association between education and health behaviors, adjusted for sex and age (Fig. 1). To further determine educational differences in the prevalence of health risk behaviors, a linear regression analysis, adjusted for sex and age, was used (Table 3).

**Table 2:** Characteristics of the study population; n (%)

Characteristics	Men	Women	Total	P-value
N (%)	13 122 (46.8)	14 925 (53.2)	28 047 (100)	
**Age groups (%)**				
18–24 years	1 274 (9.7)	1 895 (12.7)	3 169 (11.3)	
25–44 years	3 957 (30.2)	5 223 (35)	9 180 (32.7)	
45–66 years	5 813 (44.3)	6 213 (41.6)	12 026 (42.9)	
67–79 years	1 885 (14.4)	1 487 (10)	3 372 (12)	
80 + years	193 (1.5)	107 (0.7)	300 (1.1)	
**Education (%)**				
Low education	1 660 (12.7)	1 673 (11.3)	3 333 (11.9)	***
Middle education	5 574 (42.7)	5 514 (37.1)	11 088 (39.7)	***
High education	5 822 (44.6)	7 680 (51.7)	13 502 (48.4)	***
**Health behaviors (%)**				
High physical activity	4 645 (35.7)	5 232 (35.3)	9 877 (35.5)	n.s.
Healthy diet, total	1 022 (7.8)	2 283 (15.3)	3 305 (11.8)	***
Vegetables, daily	3 456 (26.4)	6 714 (45.1)	10 170 (36.4)	***
Fruit and berries, daily	2 788 (21.3)	5 382 (36.1)	8 170 (29.2)	***
Fish, ≥ 2 times per week	5 577 (42.6)	6 271 (42.1)	11 848 (42.3)	n.s.
Non-smoker	11 032 (84.3)	12 706 (85.4)	23 738 (84.8)	**
Low alcohol consumption	8 304 (63.7)	11 788 (79.5)	20 092 (72.1)	***
**Number of health risk behaviorsª (%)**				
0 behaviors	357 (2.8)	882 (6)	1 239 (4.5)	***
1 behavior	2 746 (21.2)	3 913 (26.6)	6 659 (24.1)	***
2 behaviors	5 994 (46.4)	6 984 (47.5)	12 978 (47)	n.s.
3 behaviors	3 083 (23.9)	2 430 (16.5)	5 513 (19.9)	***
4 behaviors	745 (5.8)	503 (3.4)	1 248 (4.5)	***

n.s. non-significant, **p < 0.01, ***p < 0.001 between sex. ª Health risk behaviors; defined as the proportion of participants not meeting the recommendations of one or more health behaviors

**Table 3:** Number of health risk behaviors by level of education (%)

Number of health risk behaviors, (N)	Education level, N (%)		
	Low education	Middle education	High education
0	100 (3.1)	378 (3.5)	758 (5.7)
1	623 (19)	2295 (21)	3715 (27.9)
2	1479 (45.2)	5088 (46.5)	6363 (47.8)
3	826 (25.2)	2551 (23.3)	2116 (15.9)
4	148 (7.5)	626 (5.7)	371 (2.8)

To examine clustering patterns, we first calculated the observed and expected prevalence for all 16 possible combinations of health behaviors (Table 4) [30]. Observed prevalence was calculated as the number of participants that did or did not meet the recommendations of each health behavior, divided by the total number of participants (e.g., the proportion not meeting the recommendations for physical activity, but who met the recommendations for diet, tobacco use, and alcohol consumption). The expected prevalence for single health behaviors was calculated as the proportion of participants not meeting a specific health behavior multiplied by the proportion of participants who met all the remaining health behaviors. The expected prevalence of multiple health behaviors was calculated by multiplying the proportion of participants that did not meet the recommendations for a specific combination of health behaviors with the proportion of participants that met all the remaining health behaviors [30].

**Table 4:** Observed and expected prevalence of health risk behaviors, individually and combined¹

Number of health risk behaviors	Physical activity		Diet	Tobacco use	Alcohol	O (%)	E (%)	O/E	95% CI
4		X	X	X	X	4.52	2.42	1.87	(1.79–1.95)
3		X	X	X	-	6.14	6.22	0.99	(0.95–1.02)
		X	X	-	X	12.20	13.51	0.90	(0.88–0.92)
		X	-	X	X	0.14	0.32	0.44	(0.31–0.57)
		-	X	X	X	1.47	1.33	1.10	(1.01–1.20)
2		X	X	-	-	35.61	34.81	1.02	(1.01–1.03)
		-	-	X	X	0.11	0.18	0.62	(0.40–0.83)
		X	-	X	-	0.46	0.83	0.55	(0.45–0.65)
		-	X	-	X	7.84	7.42	1.06	(1.02–1.09)
		X	-	-	X	0.88	1.80	0.49	(0.43–0.55)
		-	X	X	-	2.06	3.42	0.60	(0.56–0.65)
1		X	-	-	-	4.58	4.63	0.99	(0.95–1.03)
		-	X	-	-	18.43	19.13	0.96	(0.95–0.98)
		-	-	X	-	0.29	0.45	0.63	(0.49–0.77)
		-	-	-	X	0.80	0.99	0.81	(0.71–0.91)
0		-	-	-	-	4.48	2.54	1.76	(1.69–1.84)

E: expected prevalence; O: observed prevalence; X: nonadherence to the recommendations; -: adherence to the recommendations. O/E; observed to the expected prevalence, where O/E indicates clustering of health behaviors. ¹Note: missing data: 410. The final sample included in this analysis is *n* = 27,637

Further, the observed to the expected ratio (O/E) was calculated for the 16 combinations of health behaviors, as described previously [31]. O/E above one indicates a positive association, which means the co-occurrence of certain combinations of behaviors is higher than expected, assuming there was no association between the included behaviors. 95% confidence intervals were calculated using bootstrap techniques and were used to assess whether the accumulation was significant in the different combinations. All statistical analysis were carried out using IBM SPSS Statistics (version 25) and Stata V14 (Stata, College Station, TX). Figure 1 was created in Microsoft Office Excel. The significance level was set to p < 0.05.

## Results

Table 2 shows the characteristics of the study population, prevalence, and co-occurrence of health behaviors, stratified by sex. The sample consists of more women, individuals with higher education, and adults aged 25–66 years. The proportion of individuals meeting the recommendations for physical activity, diet, tobacco use, and alcohol consumption were 36%, 12%, 85%, and 72% respectively. Approximately 24% of the study population (30% of the men and 20% of the women) had three or four health risk behaviors at once and only 4.5% of the study population met all the four health behavior recommendations. Furthermore, women were more likely than men to meet the recommendations for diet, tobacco use, and alcohol consumption.

Figure 1 shows the proportion meeting the recommendations for each of the four health behaviors within educational classes, adjusted for sex and age. Individuals with middle and high education had 23% (OR: 1,23, 95% CI: 1,130-1,340) and 52% (OR: 1,52, 95% CI: 1,403-1,655) higher odds of meeting the recommendations for physical activity, respectively, compared to those with low education. For diet, individuals with middle and high education had 24% (OR: 1,24, 95% CI: 1,079 − 1,428), and 2.05 times (OR: 2,05, 95% CI: 1,795-2,340) higher odds of meeting the recommendations than those with low education. Further, individuals with middle and high education had 59% (OR: 1,59, 95% CI: 1,450-1,746) and approximate three times higher odds (OR: 2,96, 95% CI: 2,691-3,261) of meeting the recommendations for smoking, compared to individuals with low education. Alcohol consumption showed a less clear pattern, with differences only evident between the low and high education group (p < 0.001).

Table 3 shows the number of health risk behaviors by level of education. Significant differences were found in the number of health risk behaviors between different educational classes (p = 0.001). Higher education was associated with having a lower number of health risk behaviors (b: -0,182, 95% CI: -0,197, -0,168), respectively.

Table 4 shows the observed and expected prevalence (O/E) ratios of the sixteen possible combinations of the four health risk behaviors. O/E ratios ranged from 0.44 to 1.87, whereas five of the sixteen health risk behavior patterns had O/E ratios above 1, which is higher than expected based on the individual probabilities of the four health risk behaviors alone. The proportion of individuals having zero and four health risk behaviors simultaneously showed the largest deviation from expected proportions (1.76 (1.69–1.84)), and (1.87 (1.79–1.95)) respectively. Other health risk behavior patterns also clustered; meeting the recommendations for physical activity, but not meeting the recommendations for diet, tobacco use, and alcohol consumption (1.10 (1.01–1.20)); meeting the recommendations for tobacco use and alcohol consumption, but not meeting the recommendations for physical activity and diet (1.02 (1.01–1.03)); and meeting the recommendations for physical activity and tobacco use, but not meeting the recommendations for diet and alcohol consumption (1.06 (1.02–1.09)), respectively. The three most common health behavior patterns consisted of 66% of the study population, covering the proportion not meeting the recommendations for both physical activity and diet (36%), diet alone (18%), and physical activity, diet, and alcohol consumption (12%).

## Discussion

Our findings showed an association between sex, education, and health behaviors. Males and individuals with low education were at higher risk of having multiple health risk behaviors, and clustering patterns were found in five of the sixteen health behavior patterns.

The proportion that met the recommendations for physical activity, diet, and tobacco use was highest among individuals with high education, and the observed magnitudes are of relevance for public health policy, with for example individuals with high education being 52% more likely to meet physical activity recommendations and three times more likely to meet the recommendations for smoking These findings confirm previous research. According to Berrigan et al., higher education was associated with a higher proportion of individuals meeting all five health behavior recommendations (physical activity, fruit and vegetable consumption, tobacco use, alcohol, and % of fat in the diet). In addition, high education was associated with healthy behaviors in several previous studies [8, 32, 33]. The educational differences in health behaviors can possibly be explained by the fact that individuals with high education often determine access to greater opportunities and a healthier lifestyle, as well as higher incomes and safe neighborhoods [34, 35].

Further, we found that men had a greater extent of three and four health risk behaviors than women, reinforcing previous research [16, 36]. In addition, women were more likely to meet the recommendations for diet, tobacco use and alcohol consumption than men. High alcohol consumption was the health risk behavior that differed the most between men and women in this study and likewise in the literature [15, 36].

As for the two extreme patterns of behavior (both ends of the lifestyle scale) we found that meeting the recommendations for all or none of the four health behaviors were the two patterns that showed the largest deviation from expected values. Interestingly, clustering at both ends of the lifestyle scale turns out to be a consistent pattern in earlier research [15, 37]. Further, the most common health behavior pattern was to not meet the recommendations for both physical activity and diet at once, consisting of 36% of the sample, supporting previous research [14, 36].

Previous studies that analyzed the same four health behaviors (physical activity, fruit/vegetable consumption, tobacco use, and alcohol consumption) have also reported high co-occurrence of low physical activity and low fruit and vegetable consumption in their study populations [15, 16, 38]. Likewise, a systematic review confirmed that low physical activity and low fruit/vegetable consumption (alone and/or combined) were the two health risk behaviors that had the highest prevalence in two studies that included over 18,000 participants [39], whereas other studies in the systematic review found a combination of low fruit and vegetable intake and smoking as the most prevalent behavior [39]. Different from our findings, another study found co-occurrence of smoking and high alcohol consumption as the most prevalent health behavior pattern [37]. Thus, the most common health behavior patterns are not consistent but seem to vary among different populations, subgroups and between different countries. These variations demonstrate the importance of implementing tailored public health interventions designed to reach specific municipal health behavior challenges or subgroups. Another more general approach might be to encourage a healthy lifestyle as opposed to focusing on single behavior-specific changes in a population. This is mainly because a positive lifestyle change could lead to improvement in multiple other health behaviors at the same time, instead of changes in single behaviors only. Such a holistic approach may be valuable in order to provide a better overall health in the population, since individuals with multiple healthy behaviors have reduced risk of disease and premature death later in life [20, 23].

In our Norwegian study sample, 30% of the men and 20% of the women had three or four health risk behaviors at the same time. Compared to various studies from different countries, these findings are above average. In China, 10% of the men and only 0.7% of the women had three or four health risk behaviors at once [38]. In a study in Brazil, the prevalence was 18% for men and 11% for women [15], and further 16% for men and 9% for women in a Swedish sample population [33]. Moreover, only one study that observed an English adult population had close to similar results as ours, whereas 29% of the men and 24% of the women had three or more health risk behaviors at the same time [16]. However, substantial heterogeneity in operationalization of health risk behaviors hampers comparison between studies.

Strengths of the current study include the large, randomly drawn sample and the high response rate underpinning the representativeness of the findings. However, there are some limitations. The cross-sectional nature of the study hampers our ability to establish causality. In addition, self-reported health behaviors are associated with numerous biases, especially recall and social desirability bias [40]. Furthermore, the operationalization of health behaviors significantly affects the prevalence estimates and thus should be interpreted with caution.

Dichotomizing the four health behaviors used in this study had some strengths and weaknesses for the findings. First, due to insufficient response options regarding fruit and vegetable consumption (see Table 1), at least one portion of fruit/berries and one portion of vegetables daily (a total of 2 portions each day) were used as a cut-off point instead of the nationally recommended 5 portions each day [26]. This cut-off point, which together with fish intake provided one diet variable, should be taken into account when comparing our data with other studies, and in relation to adherence to public health recommendations. However, using separate recommendations for diet merged into one variable may simplify and strengthen the diet variable since nutritional epidemiology often is burdened by multicollinearity [41, 42]. Second, the dichotomization of alcohol consumption is based on a validated and reliable tool that measures high alcohol consumption (AUDIT-C) in a general population [27, 28] and is not directly comparable to the Norwegian recommended alcohol intake (20 g daily for men and 10 g daily for women) [43]. Nevertheless, reporting adherence to recommendations in general is useful from a public health perspective as our findings can be linked to national public health policy and goals, given the aforementioned limitations.

## Conclusion

We observed a gradient in meeting health behavior recommendations across levels of education, showing subgroups within the general adult population with an overall unhealthy lifestyle. It appeared that males and individuals with low education had higher risk of developing multiple health risk behaviors indicating that these groups could be targeted in future public health interventions. Nearly 24% of the study population had three or more health risk behaviors at once and five of the sixteen health behaviors patterns did cluster. Although more research is needed on both the causal relationships between socio-economic factors and health behaviors and the associations between clustering of these and health outcomes, we believe our findings can assist in developing informed public health interventions to combat the burden of non-communicable diseases.

## Data Availability

The Norwegian Institute of Public Health is legally responsible and the owner of the Norwegian Counties Public Health Surveys. Data may be provided from NIPH on a reasonable request.

## Declarations

### Author’s information

SBK is a MSc in public health science, BHH is a professor specializing in physical activity, health issues and statistics & TB is an associate professor specializing in public health nutrition.

## Abbreviations

NCD: Non-Communicable Disease
NIPH: The Norwegian Institute of Public Health
SMS: Short Message Service (text message)
FEK: Faculty Ethical Committee
NOK: Norwegian Krone
EUR: The Euro
AUDIT-C: Alcohol Use Disorders Identification Test-Concise
O/E: The Observed to Expected ratio
P: P-value
OR: Odds Ratio
CI: Confidence Interval
